# Targeting energy metabolism via the mitochondrial pyruvate carrier as a novel approach to attenuate neurodegeneration

**DOI:** 10.1186/s13024-018-0260-x

**Published:** 2018-05-24

**Authors:** Emmanuel Quansah, Wouter Peelaerts, J. William Langston, David K. Simon, Jerry Colca, Patrik Brundin

**Affiliations:** 1 Center for Neurodegenerative Science, Van Andel Research Institute, Grand Rapids, 333 Bostwick Ave, Michigan, 49503 USA; 2 KU Leuven, Laboratory for Gene Therapy and Neurobiology, 3000 Leuven, Belgium; 3 Stanford Udall Center, Department of Pathology, Stanford University, Palo Alto, CA USA; 4 Neurology, Beth Israel Deaconess Medical Center and Harvard Medical School, Boston, MA USA; 5 Metabolic Solutions Development Company, Kalamazoo, MI 49007 USA

**Keywords:** Mitochondrial pyruvate carrier, Insulin sensitizers, Neurodegeneration, Parkinson’s disease

## Abstract

Several molecular pathways are currently being targeted in attempts to develop disease-modifying therapies to slow down neurodegeneration in Parkinson’s disease. Failure of cellular energy metabolism has long been implicated in sporadic Parkinson’s disease and recent research on rare inherited forms of Parkinson’s disease have added further weight to the importance of energy metabolism in the disease pathogenesis. There exists a new class of anti-diabetic insulin sensitizers in development that inhibit the mitochondrial pyruvate carrier (MPC), a protein which mediates the import of pyruvate across the inner membrane of mitochondria. Pharmacological inhibition of the MPC was recently found to be strongly neuroprotective in multiple neurotoxin-based and genetic models of neurodegeneration which are relevant to Parkinson’s disease. In this review, we summarize the neuroprotective effects of MPC inhibition and discuss the potential putative underlying mechanisms. These mechanisms involve augmentation of autophagy via attenuation of the activity of the mammalian target of rapamycin (mTOR) in neurons, as well as the inhibition of neuroinflammation, which is at least partly mediated by direct inhibition of MPC in glia cells. We conclude that MPC is a novel and potentially powerful therapeutic target that warrants further study in attempts to slow Parkinson’s disease progression.

## Background

Parkinson’s disease (PD) is traditionally classified as a movement disorder due to the signature motor deficits (rigidity, hypokinesia, tremor and postural instability) resulting from the functional decline and loss of midbrain dopaminergic neurons. Importantly, non-motor deficits including autonomic features (e.g. constipation and urinary bladder dysfunction), hyposmia, REM sleep behavior disorder, depression, anxiety and cognitive deficits are also salient features of the disease [1, 2]. Some of these symptoms and signs even precede the onset of motor deficits, often by several years.

The etiopathogenesis of *sporadic* PD remains enigmatic. It has been suggested that up to 30% of the collective PD risk emanates from genetic risk, i.e. genetic polymorphisms in over 40 loci, several of which have been suggested to be associated with genes involved in lysosomal and immune functions [3]. The remaining risk for PD is suggested to be environmental (e.g. pesticides, dietary habits, have been implicated) and age related. In fact, ageing appears to be the most significant contributor, with more than 90% of patients being diagnosed at age 60 or above [4]. Whereas most cases of PD are sporadic, there are a number of identified dominant and recessive mutations which are associated with parkinsonism that play a causative role in 5–10% of all PD cases [5, 6]. Rare mutations are found in the α-synuclein (αSyn) gene, which encodes the major protein component of Lewy bodies, a signature neuropathological feature of PD. Other PD-associated genes are in molecular pathways involved in vesicular trafficking, lysosomal activity and protein clearance (e.g. VPS35, GBA and possibly also LRRK2) [6, 7]. Some PD-associated gene mutations (such as PINK1, Parkin and DJ-1) have been linked to mitochondrial dysfunction. Specifically, these mutations have been suggested to increase the generation of cellular reactive oxygen species [7, 8] and the selective degradation of dysfunctional mitochondria known as “mitophagy” [9, 10]. These discoveries resulted in multiple drug discovery programs directed at these specific pathways in attempts to find a disease-modifying therapy [11–14]. Interestingly, there are parallel drug discovery efforts to find disease-modifying treatments for Alzheimer’s disease and other major neurodegenerative diseases [15].

In recent years, there has been a growing appreciation of commonalities in metabolic impairments between type 2 diabetes and PD. Common metabolic abnormalities, including insulin resistance and mitochondrial dysfunction have been shown to be a part of these apparently disparate diseases [16–19]. While some studies have suggested an overlap in the incidence of type 2 diabetes and PD, possible effects of anti-diabetic drugs on PD progression remain understudied and controversial.

Nevertheless, the potential links between pathogenic mechanisms in PD and type 2 diabetes have stimulated clinical trials in PD using drugs approved for treatment of diabetes. For instance, Aviles-Olmos and colleagues demonstrated in a single-blinded trial (evaluation of most motor symptoms on a video, by a blinded neurologist) that treatment of moderately advanced PD with the once-weekly glucagon-like peptide 1 (GLP1)-agonist exenatide for 12 months led to better motor and cognitive scores in the 20 treated patients than in the 24 controls [20]. Specifically, exenatide-treated patients exhibited less motor deficits on the Movement Disorders Society Unified Parkinson’s Disease rating scale (MDS-UPDRS, part III) at two months after a” washout” of the treatment, which was the predefined primary endpoint [20]. Furthermore, exenatide-treated patients showed less cognitive decline as assessed by the Mattis dementia rating scale. Notably, when all the participants of the original cohort were followed for an additional ten months, i.e. 12 months after cessation of exenatide treatment, the significant difference between exenatide-treated and control groups still persisted [21]. In a second study, patients were injected once weekly with a slow release form of exenatide (Bydureon) for 48 weeks [22]. Similar improvements were recorded on MDS-UPDRS (part III)-defined primary endpoints (but not in Mattis dementia rating scale), with the beneficial effects persisting for at least 12 weeks after the removal of drug treatment [22]. In a follow-up analysis of secondary endpoints in the same study, significant positive effects of exenatide were noted on several observer and patient-led scales assessing mood, and these effects were not correlated to the motor improvement, suggesting that exenatide acts on multiple brain circuitries [23]. However, neither of these trials was designed to definitively determine if exenatide has a disease-modifying effect in PD, but these promising proof-of-concept results clearly suggest that a large multicenter trial is warranted.

Beside the studies on exenatide, several studies have explored whether the use of other anti-diabetic agents affect the risk of developing PD. Compared to other anti-diabetic drugs the use of insulin sensitizers, specifically thiazolidinediones (TZDs) also known as glitazones, were found to be associated with a reduced risk of developing PD by almost 30% in two reports [24, 25], although a different study did not replicate these findings [26]. There is considerable evidence that the first-generation insulin sensitizer compound pioglitazone, a TZD, is neuroprotective in cellular and animal models [27, 28]. The only prospective evaluation of this compound in subjects with PD revealed a trend towards less worsening on MDS-UPDRS (part III) primary outcome parameters over 44 weeks, although this was not significant [29]. However, there were several caveats to this particular pioglitazone clinical trial. For instance, the treatment duration was only 44 weeks, which is relatively short for detecting disease progression on the MDS-UPDRS scale, even in a well-powered study. The treatment duration is especially crucial since pioglitazone like other TZDs such as rosiglitazone is primarily an agonist of the nuclear transcription factor peroxisome proliferator-activated receptor gamma (PPARγ) and structural changes in synapses or dendritic spines, which might take long to develop, have been suggested as potential target effects of this drug in the brain.

Importantly, a new generation of insulin sensitizers directed against a novel mitochondrial target, rather than PPARγ are actively being investigated [30]. The newly identified mitochondrial target of these new compounds is the mitochondrial pyruvate carrier protein (MPC) [26–28]. The therapeutic potential of one of these new insulin sensitizers has been demonstrated in both in vitro and in vivo models of PD and the data support the concept that PD-relevant pharmacology is achieved through partial inhibition of the MPC and attenuation of pyruvate entry into the mitochondria. Targeting the MPC potentially causes disease-modifying effects [31]. In this manuscript, we will briefly discuss the MPC, the concept behind the development of new insulin sensitizers that target the MPC, and data supporting the clinical testing of the MPC targeting compound MSDC-0160 in PD.

### The mitochondrial pyruvate carrier: An essential player in cellular metabolism

Tight regulation of cellular metabolism is required for maintaining cellular homeostasis and cell survival. Glucose, amino acid and fatty acid metabolism are crucial cellular metabolic processes with pyruvate being a major metabolic component in these pathways. The breakdown of glucose in the cytosol via glycolysis is an important means by which the 3-carbon product pyruvate is generated. Under aerobic conditions, pyruvate can be converted into acetyl-coenzyme A (acetyl-CoA), an important molecule that enters the citric acid cycle for the generation of ATP and other high energy reducing molecules that power the electron transport system and maintain cellular energy homeostasis [32, 33]. The conversion of pyruvate into acetyl-CoA occurs in the mitochondrial matrix. Hence, to gain entry into the matrix, pyruvate requires transport across the outer membrane, intermembrane and the inner membrane of the mitochondria. While pyruvate gets across the outer mitochondrial membrane (via porins or non-selective channels) and intermembrane space with relatively less burden, the passage across the inner membrane for entry into the matrix is restricted. Like other metabolites, pyruvate needs a specific transporter to ferry it across the inner membrane into the matrix. Evidence from two labs demonstrate that two mitochondrial inner membrane proteins are responsible for transporting pyruvate into the mitochondrial matrix [34, 35]. These proteins have now been named the mitochondrial pyruvate carrier proteins 1 (MPC1) and 2 (MPC2), previously known as BRP44L and BRP44, respectively (for review, see [32, 33]).

Deletion of MPC1 in yeast and mammalian models leads to defective mitochondrial pyruvate uptake and oxidation, culminating in cellular pyruvate accumulation [31, 35]. In contrast to the effect on pyruvate accumulation, knockout of MPC1 or MPC2 causes reductions in acetyl-CoA and citric acid intermediates in some cell models [35]. Constitutive knockout of MPC2 leads to embryonic lethality, but tissue-specific knockouts are being used to evaluate the importance of MPC in various tissues [36]. The data from these genetic models therefore support the notion that MPC1 and MPC2 are essential for pyruvate transport and metabolism and that changes in the expression or activity of these proteins affect mitochondrial metabolism. Structural studies support this concept and have shown that MPC1 and MPC2 form a complex that is required for transporting pyruvate across the mitochondrial inner membrane into the matrix [37, 38]. The advances made on MPC genetics have revealed what happens when this protein is lost or is aberrant in humans. Of note, mutations in the *MPC1* gene have been found in some individuals with defective mitochondrial pyruvate oxidation, hyperpyruvatemia and lactic acidosis [35, 39]. These findings demonstrate the clinical relevance of MPC and pyruvate transport. In addition, although MPC, to the best of our knowledge, has not been linked to PD and other neurodegenerative diseases, abnormalities in MPC activity and pyruvate transport have been strongly linked to cellular mechanisms involved in the proliferation of cancer cells (in the so-called Warburg effect) [37, 40].

Due to the importance of MPC to multiple metabolic processes, pharmacological inhibitors were pursued, initially for use in controlling cell proliferation in cancer models [32, 38]. Given the recent discovery that MPC is also the target of “insulin sensitizers” thiazolidinediones (TZDs) [26, 28], selective TZDs are now being evaluated in metabolic diseases such as type 2 diabetes and non-alcoholic liver disease [30, 41, 42].

### From first generation TZDs to new insulin sensitizers that target the mitochondrial pyruvate carrier

TZDs belong to a family of heterocyclic compounds with a five membered C_3_NS ring introduced in the early 1990s for the treatment of type 2 diabetes [41]. As part of the key pharmacological effects, TZDs reduce insulin resistance, which is a primary contributor to the development of type 2 diabetes. In people with insulin resistance, the cells fail to produce a normal response to insulin. Insulin is produced in the body when glucose is released into the bloodstream following digestion of carbohydrates. Under normal conditions, the insulin produced triggers a feedback response in which cells take up glucose to be broken down into pyruvate that subsequently enters the citric acid cycle for ATP generation [43]. This ATP generation prevents the cells from breaking down fat for ATP production and also reduces circulating blood glucose to the normal range. In contrast, under insulin resistant conditions cells fail to take up glucose from the circulation, therefore blood glucose reaches abnormally high levels. The situation is further worsened when pancreatic beta cells initially increase insulin production and eventually fail, leading to type 2 diabetes. Damaged and dysfunctional mitochondria have been implicated in insulin resistance. Mitochondrial abnormalities often lead to build-up of reactive oxygen species (ROS), which further contributes to insulin resistance [43, 44]. Also autophagy and insulin sensitivity have been linked, with enhanced autophagy leading to improved insulin sensitivity in some mouse models [44].

First generation TZDs (“insulin sensitizers”) were introduced to improve insulin sensitivity in type 2 diabetes. Initially, TZDs were thought to act only by directly activating the nuclear transcription factor PPARγ [42, 43]. Once activated, PPARγ stimulates the transcription of some “metabolism-associated” genes while repressing others. Clinical studies demonstrated that TZDs improve whole-body insulin sensitivity in type 2 diabetes patients, and therefore they were approved for the treatment of diabetes [44, 45]. However, troglitazone, the first TZD to be introduced in 1997, was subsequently found to be uniquely associated with idiosyncratic hepatotoxicity and was withdrawn from the market [45]. The TZDs rosiglitazone and pioglitazone had no known hepatic side effects but were linked to edema, bone loss, plasma volume expansion that can worsen congestive heart failure and weight gain [42, 46, 47]. These side effects, which arise as a result of the direct activation of PPARγ, have substantially limited the clinical use of these TZDs.

Over 20 years of clinical research has revealed that pioglitazone, the weaker of the two PPARγ-activating TZDs used clinically [48] has a better clinical profile than rosiglitazone [49, 50]. Although TZD use has been limited over questions of cardiovascular safety, long term treatment has been suggested to reduce the risks of heart attack, stroke [51] and dementia [52]. When PPARγ was implicated in the side effects of TZDs, research was launched into the precise mechanism of action of TZDs and new generations of TZDs that might bypass PPARγ but still retaining the clinical benefit, were developed. Chen and colleagues demonstrated that TZD analogues that do not bind or directly activate PPARγ can still exhibit similar insulin sensitizing pharmacology as rosiglitazone and pioglitazone. Moreover, these new TZD analogues still exerted the same effects on the expression of metabolic enzymes in hepatocytes derived from liver-specific PPARγ knockout mice [53]. This finding was important as it suggested that direct activation of PPARγ, thought to be the primary target of the first-generation TZDs [48, 54], was not be required to achieve the well-known pharmacological actions of TZDs [53]. Thus, other mechanisms could be involved in the insulin-sensitizing effects of TZDs, an insight that provided a new route for the development of novel anti-diabetic drugs [41].

As previously hinted, it eventually emerged that the primary target for TZDs is MPC [34, 35, 47]. It is now known that while TZDs vary dramatically in their ability to directly activate PPARγ they can all attenuate the transport of pyruvate through MPC [55, 56]. The protoype MPC-targeting drug MSDC-0160 not only improves insulin-sensitivity, but has also is beneficial is several cell and animal models of PD (discussed below).

### Novel targets of the mitochondrial pyruvate carrier: The insulin-sensitizing MSDC-0160

#### Development, clinical effects and safety profile in disease models and individuals with metabolic disorders

The adverse effects associated with PPARγ activation and the discovery that the beneficial effects of TZDs do not require PPARγ prompted the development of novel drugs with little affinity for PPARγ. Two such drugs are MSDC-0602 and MSDC-0160. Several studies have shown that MSDC-0160 is an excellent insulin-sensitizer, despite the drug and its major hydroxymetabolite having extremely low affinities for PPARγ (over 250- and 50-fold lower, respectively) compared to rosiglitazone (Table 1) [30], making it a ‘PPARγ-sparing’ drug. Instead MSDC-0160 enhances insulin sensitivity by inhibiting the MPC complex. In a Drosophila model of insulin resistance, treatment with MSDC-0160 significantly enhanced insulin sensitivity [47]. This beneficial effect of the drug was suggested to be mediated by the MPC complex, as deletion of the Mpc1 orthologue resulted in a loss of the drug effect in the Drosophila model [47]. Further evaluations indicated that the drug’s action was, at least in part, related to the alterations in pyruvate, a key substrate in the citric acid cycle. These findings place insulin sensitizers such as MSDC-0160 at the heart of the metabolism of carbohydrates, amino acids and fatty acids [47]. Notably, dysfunctions of such cellular metabolic pathways are implicated in the pathophysiology of insulin resistance [57, 58].

**Table 1:** Comparison of the half-life and PPARγ binding affinities of MSDC-0160 and commonly used TZDs

*Drug*	*PPAR*γ *binding IC*_*50*_*(μM)*	*MPC binding (μM)*	*C*_*max*_ */half life*
Rosiglitazone	0.112	1.1	1 µM/3–4 h
Pioglitazone	1.535	1.2	4 µM/5–8 h
MSDC-0160	31.648	1.2	12 µM/12 h

Based on the attractive pharmacological profile of MSDC-0160 in preclinical models, it was entered into clinical trials. A double-blind, randomized phase II b clinical trial over three months in individuals with type 2 diabetes explored three exposures of MSDC-0160 as compared to pioglitazone. In this trial, MSDC-0160 reduced plasma glucose and elicited similar beneficial effects as pioglitazone, but without the undesirable side effects [52]. Importantly, the trial with MSDC-0160 also demonstrated that the drug can be dosed to a 50% higher circulating exposure. The highest MSDC-0160 dose explored produced an area under the curve (AUC) of 90,000 ng.hr/ml as compared to 60,000 ng.hr/ml for 45 mg pioglitazone (parent and active metabolites). Table 1 compares the IC_50_ for the effects of the compounds on binding to PPARγ versus MPC, along with the C_max_. Based on these data, a phase II a clinical trial was conducted in subjects with Alzheimer’s disease.

A phase IIa study in non-diabetic subjects with mild to moderate Alzheimer’s disease demonstrated that treatment with 150 mg/day MSDC-0160 for three months resulted in significant changes in the pattern of ^18^F-2deoxyglucose uptake on positron emission tomography (PET) scans as compared to the placebo-treated subjects [59]. Analysis of these PET scans suggested increased glucose uptake in brain regions known to be affected in Alzheimer’s disease suggesting that oral treatment was having central (possibly neuroprotective) effects. Attention was then turned to the study of MSDC-0160 in models of PD where the mechanisms involved could be probed in more detail [60].

#### Neuroprotective effects of MSDC-0160 in cell and animal models of Parkinson’s disease

**Fig. 1:**
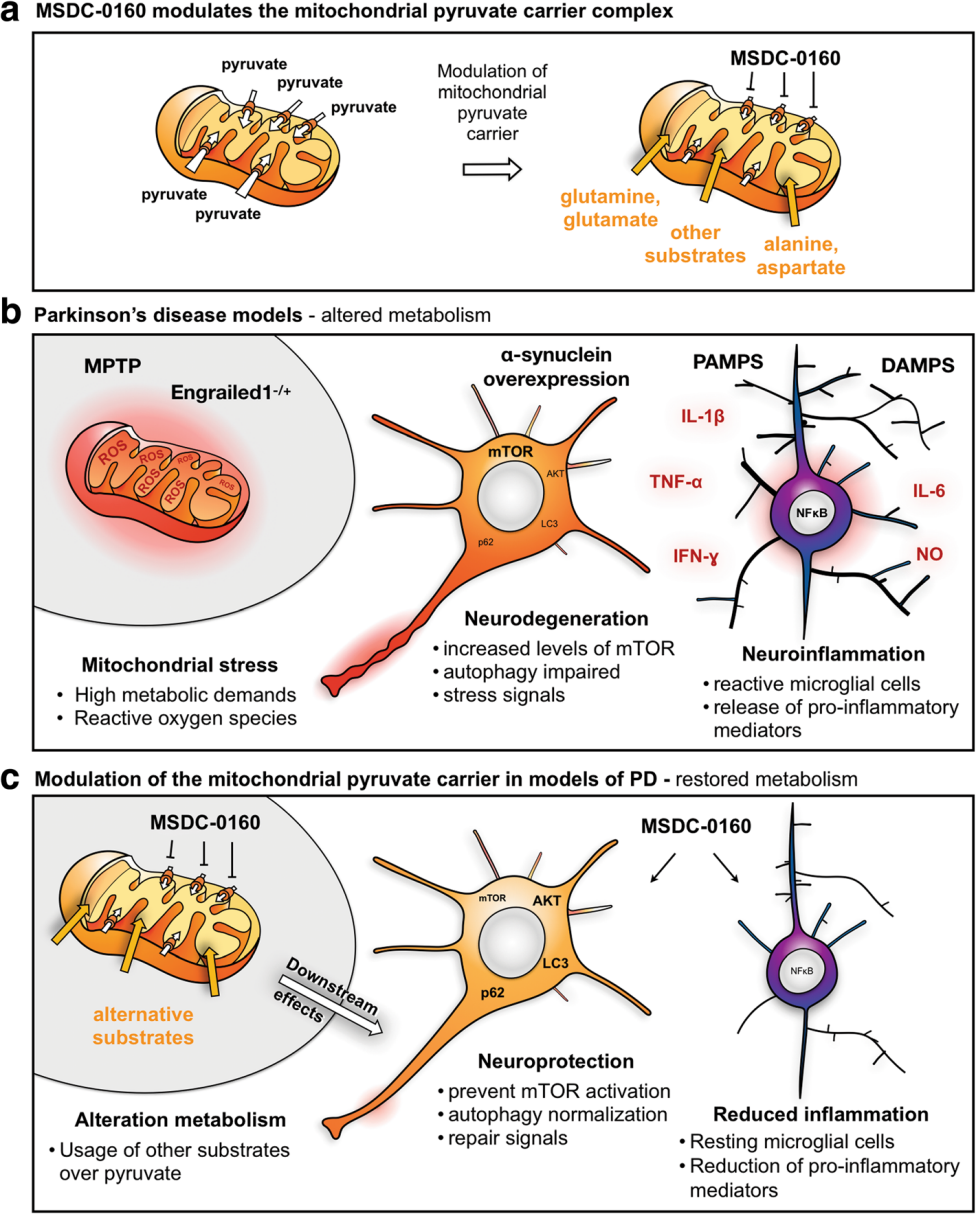
Attenuation of mitochondrial pyruvate transport by MSDC-0160 restores metabolic pathways in neurons and glial cells. **a**) MSDC-0160 slows the uptake of pyruvate into mitochondria by modulating the mitochondrial pyruvate carrier complex. This lowers the direct usage of pyruvate as a substrate for the tricyclic carboxylic acid cycle (TCA) and perhaps lowers the production of harmful reactive oxygen species (ROS); **b**) Different insults (MPP^+^, En1^−/+^ and ɑ-synuclein overexpression) producing Parkinson-related pathophysiology in animal models result in neurodegenerative changes in neurons and induce reactive microglial cells. These responses involve relative activation of mTOR activity and changes in AKT activation. In sensitive neurons, this is associated with reduced autophagy and increased cell death. Similar changes in mTOR and AKT are observed in microglial cells correlating with increased inflammation including increases in inducible nitric oxide synthase and cytokine release. During the process of neurodegeneration, pathogen-associated and damage-associated pattern molecules (PAMPS and DAMPS) activate microglial cells resulting in the release of pro-inflammatory molecules; **c**) Attenuation of pyruvate uptake by MSDC-0160 has direct effects on both neurons and microglia counteracting the effects of the environmental and genetic insults. The attenuation of pyruvate uptake by mitochondria in multiple cell types changes the metabolic balance signals in a way that attenuates the activation of mTOR, while activating the autophagic pathway. The overall protection and recovery from the Parkinson-related pathophysiology involved direct effects on both neuronal and glial cells. The direct effects of MSDC-0160 on glial cells may also indirectly affect other cell types due to the release of pro-inflammatory cytokines. Inhibiting the mitochondrial pyruvate carrier complex via MSDC-0160 restores oxidative consumption in glial cells leading to downstream alterations in the mTOR signaling pathway and a consequent reduction of pro-inflammatory molecules. This metabolic rewiring alters the activation state of microglial cells which is beneficial for limiting the neurodegenerative process. The study of interactions between neuronal and glial cells, as well as cells within the central and peripheral nervous system may aid in understanding the impact of metabolic modulators on these processes and help in the design of clinical trials and novel drugs

Recently, we demonstrated that MSDC-0160 attenuated neurodegeneration in multiple cell and animal models of PD by a process that includes autophagy augmentation and inflammation reduction [60]. These effects occurred both in genetic and neurotoxin-based PD models. These actions of MSDC-0160 involved effects on both neurons and glial cells (Fig. 1) [60].

Specifically, the effect of MSDC-0160 was evaluated in models induced by 1-methyl-4-phenyl-1,2,3,6-tetrahydropyridine/1-methyl-4-phenylpyridinium (MPTP/ MPP^+^) and in genetic models with hemizygous loss of Engrailed 1 (En1) in mice or neuronal overexpression of α-synuclein in *Caenorhabditis elegans* (*C. elegans*) [60]. MSDC-0160 treatment protected against MPTP-induced loss of tyrosine hydroxylase (TH)-immunoreactive neurons, both in those derived from Lund human mesencephalic (LUHMES) cells and in TH-immunoreactive mouse primary mesencephalic neurons. In addition to protecting dopamine neurons in cell culture, the drug also reduced MPP^+^ induced dopaminergic neuron loss in vivo in *C. elegans*. Further, the effect of the drug was evaluated in mammalian models. In this regard, MPTP was utilized to induce PD-like features in mice including loss of nigral dopaminergic neurons and dopaminergic terminals in the striatum. When evaluated using multiple methods (e.g. cell counts, western blotting and neurochemistry), the toxic effects of MPTP were significantly reduced through pretreatment with MSDC-0160. A similar protective effect of the drug was observed even when it was delivered 2 days after MPTP toxicity had been induced in mice. Behaviorally, MPTP injections caused the expected impairments in locomotion (reductions in distance traveled, speed, mobility time in an open field and time spent on a rotating rod), akin to the hypokinetic syndrome of PD. MSDC-0160 treatment mitigated these behavioral impairments. Experiments in *En1* hemizygous null mice, that normally exhibit a protracted 40% loss of nigral dopamine neurons, showed that MSDC-0160 could significantly reduce nigrostriatal degeneration and attenuate the development of motor deficits [60]. Similar to the protective effect of MPC inhibition using MSDC-0160 on dopaminergic neurons, other studies have shown that MPC inhibition using UK-5099 protects cortical neurons against excitotoxic injury by modulating glutamate abundance and release [31, 61]. Given that MSDC-0160 attenuated MPC in both neurons and microglia in the PD models, it is still not clear whether the neuroprotective effect of the drug was due primarily to its action on MPC in either one of these two cell types or both. The individual far-reaching effects of MPC modulation in microglia and neurons are depicted in Fig. 1.

#### Effects of MSDC-0160 on autophagy in PD models

To understand the mechanism by which MSDC-0160 protects against neurodegeneration, we turned to the *C. elegans* PD model [60]. In this worm model, modulation of the function of the MPC1 orthologue rescued dopaminergic neurons overexpressing A53T α-synuclein (a mutation that causes autosomal dominant PD in humans). Downstream of MPC activity and crucial for autophagy is the mammalian target of rapamycin (mTOR) and its signaling pathway. Thus, we knocked down key players in the MPC and mTOR pathways using RNA interference in these worm models and then evaluated if the effect of MSDC-0160 was maintained. Using this approach, knockdown of the MPC1 orthologue, and the mTOR orthologue or its regulators such as AKT-1 and RHEB-1 prevented the neuroprotection induced by MSDC-0160, implicating the mTOR pathway as a mediator of MSDC-0160 effects. These effects were similar to what was previously published in flies, where knockdown of MPC1 or its modulation with MSDC-0160 altered AKT phosphorylation status [47]. In contrast, knockdown of the orthologue of the cellular energy sensor AMPK (AMP-activated protein kinase which is an upstream regulator of mTOR-mediated autophagy following changes in cellular energy state) did not prevent the neuroprotection induced by MSDC-0160 [60].

Complete pharmacological inhibition of MPC using UK-5099 has been shown not to affect cell viability or oxygen consumption significantly and maintains a degree of ‘metabolic flexibility’ in healthy cortical neurons and astrocytes [61]. To define the effects of MPC inhibition on oxygen consumption in a setting potentially more relevant to PD, we measured the effects of MSDC-0160 on oxygen consumption in cultured dopaminergic neurons exposed to the mitochondrial complex inhibitor MPP^+^. Under these stressful conditions, the drug normalized oxygen consumption in cells exposed to MPP^+^, indicating a direct metabolic effect of MSDC-0160 on mitochondrial respiration, upstream of the mTOR pathway. Importantly, we also established the effects of MSDC-0160 on autophagy in vivo. In our in vivo studies using the MPTP and En1 models, there were abnormalities in mTOR signaling and autophagy, reflected by increases in ratios of p-mTOR/mTOR ratio and the downstream substrate pS6/S6, as well as changes in DNA damage responses (REDD1)/β-actin ratios. The abnormally high mTOR activation in these two mouse PD models were inhibited by MSDC-0160. Most interestingly, in no case did the treatment with MSDC-0160 reduce the activity of mTOR under control additions. In other words, rather than inhibiting mTOR activation, treatment prevented the over-activation of mTOR caused by the various manipulations. In addition, the En1 mouse PD model exhibited abnormally low ratios of the autophagy markers LC3b/β-actin and p62/β-actin, which was corrected by MSDC-0160 [60]. Together, our study revealed that MPC inhibition by MSDC-0160 leads to normalization of oxygen consumption in compromised cells and it protects against neuronal loss by inhibiting mTOR and enhancing autophagy. Notably, the onset of the aforementioned effects on mitochondrial respiration was within a few minutes of exposure to MSDC-0160, while changes in autophagy pathways took more than 24 h to be apparent. Thus, it remains to be established precisely how mTOR inhibition is achieved following MPC inhibition, and the metabolic events that follow the immediate effects on mitochondrial respiration and that precede changes in the mTOR pathway still need to be elucidated. A greater understanding of these changes will solidify the MPC as a therapeutic target in PD and might reveal new molecular targets for intervention.

#### Anti-inflammatory effects of MSDC-0160 in the PD models

Neuroinflammation is considered to play a major role in the pathophysiology of PD [62, 63]. Therefore, we further assessed the effect of MSDC-0160 on neuroinflammatory markers in different PD models [60]. In both mouse models (MPTP and En1^+/−^), MSDC-0160 reduced the microglial marker ionized calcium-binding adapter molecule 1 (Iba-1), the astrocyte marker glial fibrillary acidic protein (GFAP), and the expression of the inducible nitric oxide synthase (iNOS) in the midbrain, indicative of reductions in microgliosis and astrogliosis. Further experiments using a microglial cell line (BV2 cell line) and mouse primary microglial cells revealed that MSDC-0160 prevented lipopolysaccharide (LPS)-induced nitrite production and iNOS expression. Moreover, while phosphorylated p65 were found to accumulate in the nucleus of BV2 cells exposed to LPS, MSDC-0160 prevented this nuclear accumulation, perhaps as a result of the inhibition of NFκB (a transcription factor involved in inflammatory responses). As expected from the decreases in inflammatory markers, there were also reductions in the release of proinflammatory cytokines from LPS-stimulated BV2 cells that were exposed to MSDC-0160. Specifically, MSDC-0160 reduced LPS-induced release of the cytokines IL-1β, TNF-α and IL-6, consistent with an inhibition of neuroinflammation (Fig. 1b-c).

From a mechanistic point of view, it is clear that glial cells and neurons communicate in vivo, and it is possible that MSDC-0160 could have influenced either neuronal survival or glial activation states indirectly. However, in vitro experiments where neurons and glia are cultured separately, like those discussed above, indicate that MSDC-0160 has direct effects on both cell types. In addition, application of MSDC-0160 to LPS-treated BV2 cells also restored mitochondrial oxygen consumption back to normal levels within minutes of the drug being added to the culture [60]. Notably, in the absence of cell stressors or genetic changes we observed no measurable effects of MSDC-0160 on the different markers we monitored in either neurons or BV2 cells, or in the mouse models in vivo. This strongly suggest that modulation of the activity of MPC, a pharmacology that also improves insulin sensitivity [53], directly impacts the pathophysiology relevant to PD in both glial cells and neurons without significantly altering their normal biology. Therefore, MPC appears to be a particularly attractive molecular target for disease modification in PD.

### General cellular mechanisms associated with mitochondrial pyruvate carrier modulation and MSDC-0160 action

As discussed above, the mitochondrial pyruvate complex subunits, MPC1 and MPC2, are well conserved such that the human proteins can completely substitute for the yeast proteins in yeast knockouts [34, 35]. A growing body of literature is defining the function of this protein complex and several reviews of the current understanding are available [33, 64]. One can imagine that since these proteins are so well conserved, it is likely that the complex, as a major entry point of carbohydrate metabolism into the mitochondrion, may have evolved to control multiple inputs for the coordination of cellular respiration and metabolic processes. Thus far, it is unknown whether any human mutations or abnormalities in the MPC are associated with clinical PD or other neurodegenerative disorders. Interestingly, constitutive knockout experiments in mice have shown that *Mpc2* gene disruption results in the death of these animals between embryonic days 11 and 14 [36]. In contrast, selective knockdown of these proteins in cultured cells does not appear to have any major adverse effects on cell viability [31]. Despite the tolerance of MPC loss in cultured cells in both knockdown studies and experiments with the irreversible MPC inhibitor UK-5099, there is evidence indicating that inhibition of pyruvate entry at this site results in changes in the metabolism of amino acids that supply alternative sources of carbon to the mitochondrial metabolic pathway [31, 65]. This suggest that some sort of metabolic rewiring takes place to compensate for the loss of pyruvate entry into the mitochondria via the MPC complex and that mitochondrial metabolism can be reprogrammed to use alternative carbon sources for respiration.

**Fig. 2:**
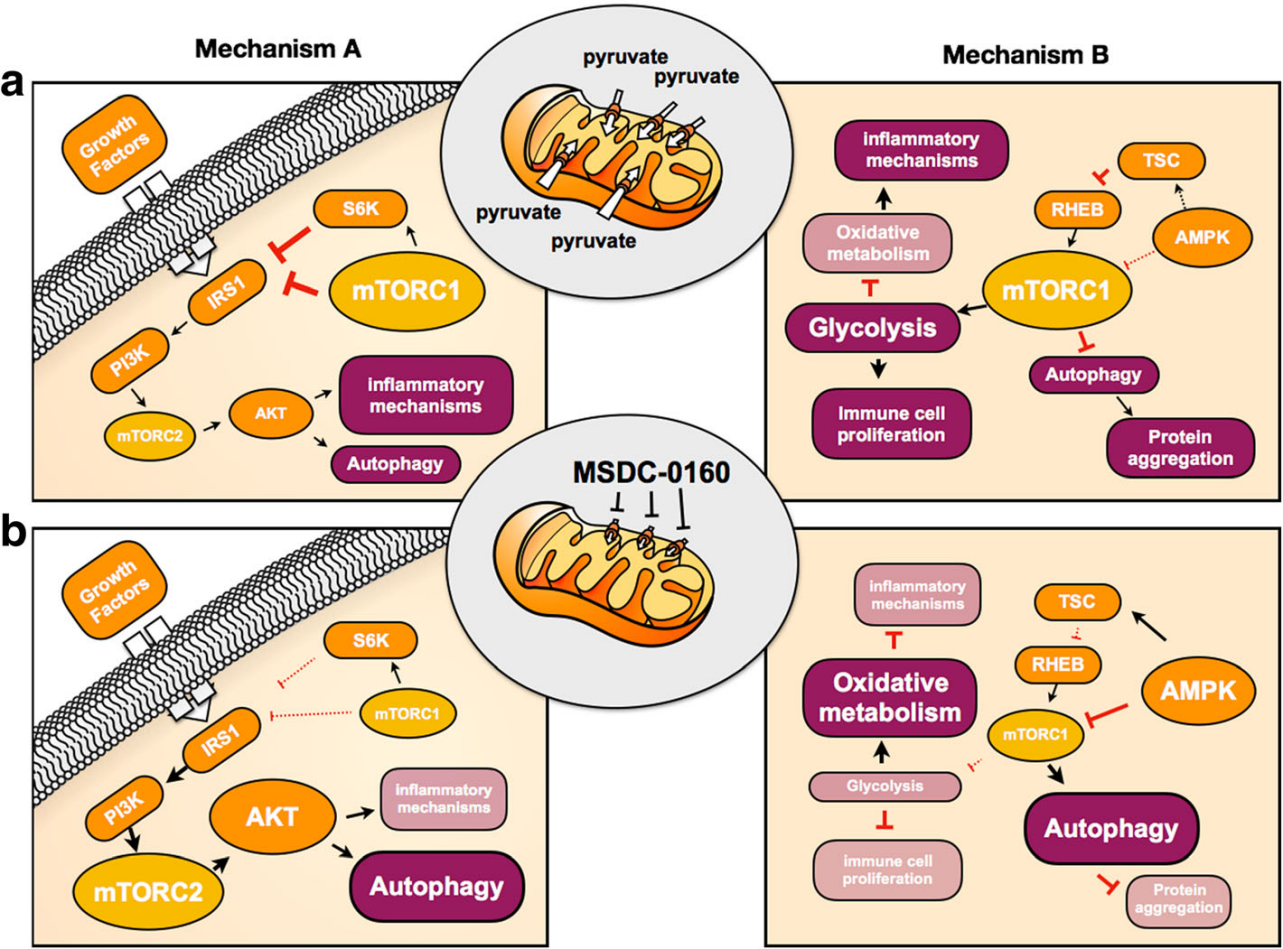
Mechanisms potentially underlying the beneficial effects of MSDC-0160. The drug’s action in Parkinson’s disease may include metabolic rewiring following inhibition of pyruvate uptake and downstream effects on mTOR and its associated pathways. We propose two hypotheses (mechanism A, left column and mechanism B, right column) to explain the observed effects of MSDC-0160. Mechanism A: (**a**) Activated mTORC1 inhibits insulin receptor substrate (IRS1) through a phosphorylation of serine residues. This inhibition of IRS1 results in dampened autophagy and enhanced inflammatory mechanisms; (**b**) Inhibition of mTORC1 (by MSDC-0160) may allow IRS1 signaling which may culminate in enhanced autophagy and cytoprotection. As an alternative mechanism (Mechanism B): (a) Activated mTORC1 is known to promote anabolic processes while inhibiting catabolic processes like autophagy; (b) Conceivably, MSDC-0160 treatment may also inhibit mTORC1 through AMPK activity, which could promote autophagic mechanisms while also minimizing inflammation

A number of compensatory mechanisms have so far been suggested (see Figs 1 and 2). For instance: (a) MPC inhibition using UK-5099 has been shown to increase the utilization of glutamine and glutamate [66], which may serve as alternative carbon sources, to compensate for the limited pyruvate oxidation. Mitochondrial malic enzyme may also facilitate the conversion of glutamine-derived malate to pyruvate, thus supplying a local pyruvate pool for cellular respiration. Interestingly, Divakaruni and colleagues have shown that by increasing glutamate oxidation, UK-5099 protects against excitotoxic neuronal death [61]. This effect occurred in isolated neuronal cells, but it is likely there would be similar effects in glia and this could contribute to the effects of the drug in vivo; (b) reduction of MPC also increases the cellular accumulation of other amino acids such as aspartate and lactate, while decreasing citrate and alanine levels [31, 66] and these changes may play various roles in maintaining cellular respiration. The pyruvate-alanine shuttle, for example, is considered to be a particularly important compensatory mechanism [67, 68], since the enzyme alanine aminotransferase can facilitate the conversion of cytosolic pyruvate into alanine, which could then enter the mitochondrial matrix where it can be transaminated back to pyruvate to feed the citric acid cycle; (c) Finally, limiting MPC-mediated uptake of pyruvate increases the oxidation of some fatty acids [31], a cellular event that generates a different source of acetyl-CoA for cellular respiration. Overall, following MPC inhibition, several alternative pathways exist for ensuring the maintenance of mitochondrial respiration. It is unknown, however, which of these pathways are most significant as alternative carbon sources for respiration in neuronal cells and ensuring neuronal cell survival.

Of particular interest to neuronal cell survival is the ability of MSDC-0160 to reduce the activation state of mTOR in PD models and the consequent enhancement of autophagy [60]. Autophagy ensures the removal of damaged or unwanted cellular components, a process mediated by the lysosome. Indeed, mTOR signaling and autophagy are known to be intimately linked, and have been strongly associated with PD together with lysosomal dysfunction [6]. Several genes related to lysosomal trafficking, lysosomal function and hence autophagy [e.g. LRRK2, GBA, ATP13A2, RAB7L1, etc.] have been implicated in PD [6] and genome-wide association studies suggest that polymorphisms in loci close to genes controlling proteins in the lysosomal-autophagy pathway influence the risk for sporadic PD [69]. Chemical compounds such as the mTOR inhibitor rapamycin induce autophagy by boosting lysosomal biogenesis and protect against dopaminergic neurodegeneration in animal models of PD [70]. Importantly, removal of aggregated α-synuclein, a major player in PD or blocking the prion-like spread of aggregated α-synuclein, are considered to be some of the most promising strategies that could positively alter disease progression [71]. Indeed, autophagy induction by mTOR inhibition and restoration of lysosomal activity may be valuable approaches to restore protein homeostasis and protect against neuronal cell death.

The exact relationship between autophagy induction and the upstream mitochondrial metabolic changes induced after MPC inhibition with MSDC-0160 are not fully known. Interestingly, the cellular energy sensor AMPK is known to function as an adaptor molecule ensuring cellular energy homeostasis, stimulation of glycolysis, induction of fatty acid oxidation and autophagy activation (Fig. 2, Mechanism B) [72]. It remains to be assessed whether AMPK plays a role in linking the cellular energy state with autophagy, following MPC inhibition using MSDC-0160 in in vitro and rodent models. In any event, activated mTORC1 is known to inhibit insulin receptor substrate (IRS1) signaling, which may also decrease autophagy. Therefore, it is possible that preventing over-activation of mTORC1 (by MSDC-0160) promotes IRS1 signaling, and that this results in enhanced autophagy and cellular protection (Fig. 2, Mechanism A) [73, 74]. Another interesting line of evidence suggests that specific changes in phosphatidylinositol metabolism can selectively repress mTORC1 activity [75], and this may provide a link between cellular metabolic state and autophagy induction via mTOR. Further investigations are needed to define the exact mechanisms underlying the protective effects of MPC inhibition. Importantly, growing evidence also associates PD with epigenetic changes [76], and these epigenetic changes are likely under metabolic control. A way to shed more light on this would be to examine the impact of MPC inhibition-induced epigenetic changes on lysosomal function [6]. If MPC inhibition (e.g. using MSDC-0160 or UK-5099) causes robust epigenetic changes, they might be used as biomarkers of target engagement in future clinical trials.

MPC inhibition using UK-5099 transiently inhibits ATP generation in cultured neurons [61], which is predicted to increase mitophagy, along with other autophagic processes. Notably, dysfunction in mitochondrial quality control mechanisms including mitophagy is implicated in some genetic forms of PD. Specifically, PINK1 and Parkin, which are mutated in some autosomal recessive forms of PD, are believed to work together to promote autophagy/mitophagy, and participate in the removal of damaged mitochondria [77, 78]. Chemicals that uncouple or stress mitochondria and limit ATP production can activate PINK1/Parkin-dependent mitophagy [77, 78]. However, whether or not enhancing mitophagy by this mechanism would have beneficial effects is uncertain and requires further study.

### Clinical implications and future perspectives

Mitochondrial function is not limited to ATP generation, as they also contribute with dynamic signals that respond to changes in the surrounding environment. Inhibiting pyruvate entry into the mitochondria may rewire cellular homeostasis by changing metabolism of amino acids that supply alternative sources of carbon to the mitochondrial metabolic pathway. In particular, pyruvate entry inhibition may cause changes in glutamine and glutamate utilization, pyruvate-alanine shuttle and fatty acid oxidation, which could in turn trigger downstream changes in mTOR activation, autophagy and inflammatory pathways in neuronal and glial cells. Evidence from studies using MPC inhibitors such as the first generation TZDs, MSDC-0160 and UK-5099 indicate that MPC inhibition indeed causes significant changes in mitochondrial metabolism and cellular homeostasis [31, 60, 61]. The metabolic and cellular changes may restore abnormalities in circulating glucose levels and insulin sensitivity in individuals with type 2 diabetes that receive the first generation TZDs or MSDC-0160.

While MSDC-0160 protects against neuronal cell death in multiple PD models, through a variety of attractive mechanisms, a number of critical questions remain to be addressed before clinical trials start:

- MSDC-0160 has been shown to slow the entry of pyruvate into the mitochondria with consequential effects on limitation of mTOR activation and the restoration of autophagy. The mechanism also includes the inhibition of inflammatory cytokine release [60]. But how does the inhibition of pyruvate entry into the mitochondria rewire mitochondrial metabolism? And what precise mechanisms link the metabolic effects of the drug with its downstream autophagic and anti-inflammatory effects? Determining the full scope of metabolic events and signaling pathways that change in response to MPC inhibition, might shed further light on the mechanism(s) by which the MPC inhibition protects neurons in PD models.
- Does MPC inhibition protect against α-synuclein accumulation and its toxic effects? Do the positive downstream effects of MPC inhibition on autophagy counteract α-synuclein aggregation or slow down the prion-like spread of α-synuclein, as this might be a predicted consequence of mTOR inhibition [69, 70]? Several studies have shown that improving autophagy can reduce α-synuclein burden [79]. Therefore, preventing over-activation of mTOR through of MPC inhibition might protect against α-synuclein accumulation.
- Is the MPC inhibitor MSDC-0160 effective in reducing non-motor effects of PD? Recent research has implicated neuroinflammation as a contributor to the many non-motor signs and symptoms seen in PD [80, 81]. Therefore, the anti-inflammatory effects of MSDC-0160 in the brain might reduce these manifestations of PD.
- Are the potentially beneficial effects of MPC inhibition in the brain primarily mediated via direct actions on neurons or glia, or do they both respond favorably to MPC inhibition under conditions of cell stress?
- Are possible neuroprotective effects of MSDC-0160 secondary to metabolic effects in the periphery? While the effects on the central nervous system might result from a direct action of the drug in the brain, it might also be secondary to changes in the metabolic milieu induced by peripheral actions.
- Does MPC inhibition cause epigenetic changes? It would be interesting to define any potential MPC inhibition-induced epigenetic changes in PD models. As mentioned earlier, such epigenetic changes could constitute biomarkers of target engagement in clinical trials.
- Will MPC inhibition by MSDC-0160 have synergistic effects with other anti-diabetic drugs in PD? It is possible that combinations of drugs with metabolic actions could be additive or even synergistic. As already mentioned, the GLP1 agonist exenatide was recently suggested to modify progression of motor symptoms in PD [22]. Interestingly, treatment of diabetes with exenatide is known to work well together with the pioglitazone and delays the need for insulin replacement [82]. This raises the important question of whether MPC inhibitor MSDC-like 0160 might synergize with the effects of GLP1 agonists in the nervous system.

## Conclusion

In summary, modulating cellular metabolism appears to be a promising approach to slow PD progression. This realization comes from a convergence of information which includes understanding that there is a metabolic component to PD pathophysiology; promising clinical trials on GLP-1R agonists in PD and the realization that MPC is a novel target of new insulin sensitizers that can be targeted with clinically safe drugs that show promising effects in laboratory models of PD. We suggest that clinical studies should be initiated to determine the potential of MSDC-0160 as a disease-modifying therapy in PD. In parallel, mechanism of action studies addressing the questions outlined above may “connect the dots” and explain how modulating metabolism by MPC inhibition might promote neuroprotection. Such studies could also lead to the development of biomarkers which can be used in clinical trials and could provide insights into whether combination therapies with other anti-diabetic drugs are warranted.

## Abbreviations

Acetyl-CoA: acetyl-coenzyme A
AUC: Area under the curve
En1: Engrailed 1
GLP-1: Glucagon-like peptide 1
LUHMES: Lund human mesencephalic
MDS-UPDRS: Movement disorders society unified Parkinson’s disease rating scale
MPC: Mitochondrial pyruvate carrier
MPTP/ MPP^+^: 1-methyl-4-phenyl-1,2,3,6-tetrahydropyridine/1-methyl-4-phenylpyridinium
mTOR: Mammalian target of rapamycin
PD: Parkinson’s disease
PET: Positron emission tomography
PPARγ: Peroxisome proliferator-activated receptor gamma
ROS: Reactive oxygen species
TCA: Tricyclic carboxylic acid cycle
TH: Tyrosine hydroxylase
TZDs: Thiazolidinediones
